# On the Separation of the Neck of Polypus of the Uterus, after the Removal of the Body of the Tumor

**Published:** 1826-11

**Authors:** Thomas Arthur Stone

**Affiliations:** Member of the Royal College of Surgeons; Surgeon to the Brownlow-street Lying-in Hospital, &c.


### POLYPUS OF THE UTERUS.

*On the Separation of the Neck of Polypus of the Uterus, after the Removal of the Body of the Tumor.* By Thomas Arthur Stone, Member of the Royal College of Surgeons; Surgeon to the Brownlow-street Lying-in Hospital, &c.

The relation of individual facts may perhaps appear in themselves unimportant, and deserving of but little attention; but, as all our knowledge upon every subject must depend upon an accumulation of such facts, it will, when attentively considered, be found of great moment to record whatever may add to our stock of information on any particular disease, or may tend to show the methods which is instituted by nature to free the body from complaint. It is on this account that the writer of the present paper has been induced to offer the following remarks, in which he will endeavour to prove

2

that, after the removal of firm polypous tumors, *attached by a narrow neck* to the uterus, although the greater portion, or the whole, of the peduncle by which they were connected should remain above the ligature; yet that this will be separated by the action of the absorbents of the uterus, the organ itself being subsequently left in a healthy state.

The different diseases by which the uterus is affected are now so much better understood,—their diagnostic marks so much more clearly established,—and their method of treatment, in consequence, so much improved, that no apology will be required at the present day for bringing this subject forward.

This separation of the neck of the polypus from its attachment, has been stated above to occur when the tumor is firm, and attached by a narrow neck: it will not, however, be found to take place in those cases where the tumor is of softer consistence, and where the attachment to the uterus is nearly of the same diameter with the tumor itself. Here it appears that the whole or the greater part of the uterus is involved in the disease; and, although a tumor of a very large size should be removed, yet, in the course of an exceedingly short time, another of equal dimensions will be found to have been protruded from the uterus into the vagina, occupying its place.

In the year 1816, a large tumor, resembling polypus, but softer in consistence, and having an attachment to the uterus of nearly equal breadth with the tumor itself, was removed by ligature from a lady, of about thirty-six years of age. The symptoms disappeared; but in the course of eighteen months they returned, when it was ascertained, by examination, that another tumor of the same consistence, and what at this time nearly filled the vagina, had protruded through the os uteri. This was also removed, and the symptoms again disappeared. In less than four months, however, they returned; another polypoid tumor, of the same size and character, having descended into the vagina. A ligature was applied round its neck, and in the course of a few days it came away; but, in less than a fortnight afterwards, the symptoms, which had at first diminished, although they never disappeared entirely, having again become severe, an examination was made, and another tumor, equally large and soft, was again discovered, filling completely the vagina. A ligature was also applied round this tumor. The neck of the tumor was soon cut through, and it came away in about four days, but the patient did not long survive.—The first of these tumors, which was removed in the year 1816, and the two last, were preserved, and are now in the collection of the author of this paper.

In such a case, it would *certainly* be right to remove the tumor, although we may expect a recurrence of the complaint, in the same way that it would be right to remove the cauliflower excrescence of the uterus, *when this has attained a very large size,* and, by the quantity of the watery discharge which it occasions, is threatening the life of the patient, although we are equally sure that the disease will return. In neither case do we expect to free the patient from her complaint, yet in both we may be able to prolong her existence.

Where, however, the tumor of polypus is firm in substance, and adheres by a narrow neck, then we may venture to promise a perfect removal of the disease, and we may be enabled to give great comfort to the mind of the patient, by pointing out to her that there is no fear of its recurrence. It has happened to the writer of this paper to be engaged in the removal of many polypi from the uterus; and it was observed by him, after the application of a ligature round the neck of the tumor, that in some instances, on the tumor coming away, the neck was only partially cut through at the part where the ligature was applied; and that, in these cases, the tumor had been separated from its attachment by the action of the absorbents, so that a portion, which was situated above the application of the ligature, came away. Of this occurrence a preparation is in the possession of the author, where the polypus is suspended in the spirit by the very ligature which was applied; which, of course, could not have happened, had the ligature entirely cut through the neck of the tumor.

The following case, also, which occurred in a patient of the St. George's and St. James's Dispensary, and on whom the operation was performed by my friend Mr. Blagden, in presence of Mr. Jeffreys and myself, will also illustrate this point.

Mrs. Buckle, aged forty-seven, had been suffering from discharge, occasionally coloured, for two years. It sometimes happened that large coagula of blood were voided. The menstruation had ceased for about a year before the discharge occurred. On examination, a polypus, about the size of a plover's egg, was found in the vagina; the neck being surrounded by the os uteri. On the 8th of August, 1819, a ligature was passed round the tumor, and fastened by means of the common canula. On the two succeeding days it was tightened; and on the third the tumor came away with the canula, the ligature still remaining upon the neck of the tumor, and there being a portion, of the extent of half an inch, above that point of the peduncle round which the ligature had been applied. In the course of a short time the discharge ceased, and the woman recovered. At the end of a month, I examined the os uteri, which appeared in a perfectly natural state.

In this case it is evident that the separation of the tumor from its attachment was influenced by the application of the ligature. But, as in other cases it is found that the neck of the tumor is cut through, and that in consequence the polypus is separated at the point where the ligature is applied, and not from its attachment to the uterus, a question next arises as to what becomes of the portion which is above the ligature in these cases: whether this is capable of giving rise to another polypus or not. This question appears to be set at rest by the following case.

A poor unmarried woman came to town for medical advice in 1817. She had laboured for many years under profuse discharge from the vagina, the cause of which had not been ascertained. She applied to Mr. Charles Clarke, for his opinion, and he discovered what was the real nature of her disease,—a large polypus, as big as a child's head at birth. At this time she was extremely reduced, being almost entirely drained of blood, and suffering from symptoms of dropsy. The polypus was removed; but the woman was attacked by symptoms of peritoneal inflammation, and being unable, from her debilitated state, to bear the necessary remedies, she died. On examining the body, I found in the abdomen strong traces of peritoneal inflammation. Having brought away the uterus, in order to examine it more leisurely, I found the os uteri somewhat more dilated than usual, but otherwise in a natural state. The neck of the tumor, which was of a very small size compared with the tumor itself, (it having been contained in the uterus, whilst the tumor was allowed to expand in the vagina,) was still attached to the fundus of the uterus, but the point of attachment was exceedingly small, being very much thinner than any other part of the neck of the tumor, and nearly separated from the uterus; the absorbents having been excited to action by the removal of the bulk of the tumor.—The preparations of the polypus and of the uterus are in the author's collection.

It appears, then, from the cases which have been detailed, that, after the application of a ligature to a polypus which is firm in substance and has a narrow neck, the portion above the ligature is always removed by the absorbents of the uterus, this process being sometimes completed before, and at others not till after, the coming away of the ligature. But, having stated the grounds on which this opinion is founded, it is not the intention of the writer to attempt any explanation of this separation taking place. Indeed, in the present state of our knowledge, when the causes which give rise to polypi, and the manner in which they originate, are not understood, it is not likely that any explanation that would be deemed satisfactory could be offered. At the same time, however, the knowledge of the fact itself may perhaps be productive of advantage, as far as regards the prognostic to be given with respect to the return of the complaint after the application of a ligature for the removal of the *real polypus,* or the *soft polypoid* tumor from the uterus.

*26, Argyle-street; October* 1826.

